# Epiphora in lung cancer patients receiving docetaxel: a case series

**DOI:** 10.1186/1756-0500-7-322

**Published:** 2014-05-30

**Authors:** Tomoko Yamagishi, Nobuaki Ochi, Hiromichi Yamane, Satoshi Hasebe, Nagio Takigawa

**Affiliations:** 1 Department of General Internal Medicine 4, Kawasaki Medical School, 2-1-80 Nakasange, Kita-ku, Okayama 700-8505, Japan; 2 Department of Ophthalmology, Kawasaki Medical School, 2-1-80 Nakasange, Kita-ku, Okayama 700-8505, Japan

**Keywords:** Non-small cell lung cancer, Docetaxel, Epiphora, Ocular adverse event

## Abstract

**Background:**

Docetaxel is a key antineoplastic drug for treatment of non-small cell lung cancer. Ocular adverse events of docetaxel include epiphora (excess tearing) and conjunctivitis. Epiphora has been reported to be associated with canalicular and nasolacrimal duct stenosis, but it is not necessarily caused by lacrimal duct obstruction.

**Case presentation:**

We encountered three Japanese non-small cell lung cancer patients who developed epiphora after the administration of docetaxel-based chemotherapy. One patient with lacrimal puncta stenosis showed improvement with probing and irrigation. The other two patients resolved following cessation of docetaxel or administration of artificial tears.

**Conclusion:**

As epiphora can interfere with activities of daily life and negatively affect quality of life, it is important for thoracic oncologists to be aware of this adverse event.

## Background

Docetaxel is a highly effective chemotherapeutic agent for non-small cell lung cancer (NSCLC) [1]. Common adverse events are neutropenic fever, anemia, fluid retention, hypersensitivity reactions, anorexia, myalgia, mucositis, alopecia, skin and nail toxicity, and peripheral neuropathy [2]. Epiphora (excessive tearing) was reported to occur in up to 64% of breast cancer patients receiving docetaxel-based chemotherapy [3]. It has been suggested that tearing may result from canalicular and nasolacrimal duct stenosis [4]; however, Chan *et al*. [5] reported that epiphora was not necessarily caused by lacrimal duct obstruction in breast cancer patients receiving adjuvant docetaxel-based combination chemotherapy. Although docetaxel is a key antineoplastic drug for NSCLC treatment, there have been few reports regarding epiphora in patients with NSCLC. Here, we report three NSCLC patients who developed epiphora after the administration of docetaxel-based chemotherapy.

## Case presentation

### Case 1

**Figure 1:**
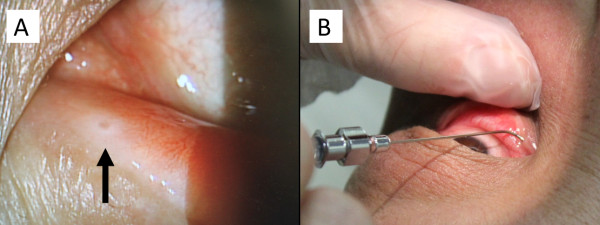
**Slit-lamp examination of the left eye and irrigation of the nasolacrimal duct.** Slit-lamp examination of the left eye revealed stenosis of the lower lacrimal puncta, which the arrow pointed to **(A)**. Probing and irrigation of the nasolacrimal duct was performed **(B)**.

A 52-year-old Japanese man was referred to our hospital for treatment of advanced NSCLC. Six years previously, he had received chest radiotherapy with concurrent chemotherapy, including cisplatin and docetaxel [6]. He had received several chemotherapeutic treatments after progression of the disease. Finally, he was treated with docetaxel monotherapy (60 mg/m^2^ on day 1 of every 21-day cycle) as seventh-line chemotherapy. The patient achieved stable disease. After a cumulative dose of 520 mg/m^2^, including first-line chemotherapy, he developed epiphora of both eyes, nail changes, and fluid retention as adverse events. Docetaxel was discontinued, but the epiphora became progressively worse, leading to a referral for ophthalmic opinion and management. On ophthalmological examination, mild stenosis of the lower lacrimal puncta was revealed by slit-lamp microscopy (Figure 1A). The epiphora improved immediately by probing and irrigation of the eye (Figure 1B).

### Case 2

A 68-year-old Japanese woman was treated with docetaxel monotherapy (60 mg/m^2^ on day 1 of every 21-day cycle) as third-line chemotherapy. The patient achieved partial response. After a cumulative dose of 720 mg/m^2^, she developed epiphora of both eyes and nail changes. The results of an ophthalmological examination were almost normal except for detection of cataracts. There was no evidence of lacrimal duct obstruction. Treatment with docetaxel was discontinued because of the adverse events. Although excessive tearing improved gradually over 2 months, the epiphora had not resolved completely at 5 months after cessation of docetaxel.

### Case 3

A 67-year-old Japanese woman was treated with docetaxel (60 mg/m^2^ on day 1 of every 21-day cycle) and bevacizumab (15 mg/kg on day 1 of every 21-day cycle) as tenth-line chemotherapy. Six years previously, she had received chemotherapy with carboplatin and docetaxel. She complained of epiphora and discomfort of both eyes after a cumulative dose of 720 mg/m^2^ of docetaxel, including first-line chemotherapy. An ophthalmological examination revealed only dry eye, and no lacrimal duct obstruction was observed. Her ocular symptoms slowly improved with the administration of artificial tears and sodium hyaluronate. The chemotherapy was continued for disease stabilization.

## Discussion

We experienced three patients with advanced NSCLC who developed epiphora after the administration of docetaxel-based chemotherapy. Common ocular toxicities associated with docetaxel administration include epiphora and conjunctivitis [7]. Chan *et al*. [5] reported that the incidences of tearing and other eye symptoms associated with docetaxel-based treatment was 86%, suggesting that epiphora was one of the most common adverse events. Excessive tearing interferes with daily life activities such as driving, reading, and visual tasks. The frequency and severity of epiphora increased with weekly administration of docetaxel compared with administration every 3 weeks [8]. In addition, ocular toxicity occurred in patients receiving a median or mean cumulative docetaxel dose of 300 or 400 mg/m^2^ or a higher dose [9]. Cumulative doses of docetaxel in our cases were all more than 500 mg/m^2^. The relations of the patients’ docetaxel exposures to expected incidence rates were prospectively analyzed [5]. There was a trend for lower rates of tearing in patients receiving lower cumulative doses of docetaxel. However, we should be aware that the combined drug with docetaxel such as fluorouracil can affect the rates.

Canalicular stenosis associated with docetaxel is most likely caused by secretion of docetaxel in the tear film and resultant chronic inflammation of the canaliculi due to direct contact with the drug as the tears travel through the canaliculi and the nasolacrimal duct to drain into the nose [4,9,10]. Importantly, epiphora occurs either with or without the presence of lacrimal duct obstruction [5], the latter being considered reactive tearing to ocular dryness [11]. Mild to moderate epiphora may resolve without medical or surgical intervention after discontinuation of docetaxel [4]. However, delayed recognition and management of this adverse event can lead to severe lacrimal duct obstruction that cannot be managed with silicon intubation and requires more complicated surgery [4]. The use of eye drops such as artificial tears may wash out docetaxel from the ocular surface and thereby prevent the development of dacryostenosis [11].

Epiphora associated with the treatment using other taxane, paclitaxel, seems to be rare. A patient who was treated with paclitaxel for angiosarcoma of the head and neck developed epiphora [12]. A phase III study comparing the efficacy of docetaxel and paclitaxel, given either weekly or every 3 weeks, in the adjuvant treatment of breast cancer showed that grade 3 and 4 tearing was reported in 5% of patients who received weekly docetaxel versus less than 1% of patients who received docetaxel once every 3 weeks, weekly paclitaxel, or paclitaxel once every 3 weeks [13]. The study also revealed that grade 2 tearing occurred 19%, 5%, 1% and 1%, respectively. In addition, ocular toxicities in NSCLC treated with paclitaxel did not include epiphora [7]. In terms of similar effectiveness of the two taxanes for NSCLC, a patient receiving docetaxel with toxicity of epiphora may change the chemotherapeutic drug to paclitaxel.

Excessive tearing in our three cases improved gradually with timely diagnosis and management. One patient with mild punctal stenosis was improved by probing and irrigation. In the other two patients, epiphora was resolved by discontinuation of docetaxel or use of artificial tears. When the symptoms do not resolve, epiphora has a substantial negative impact on the patient’s quality of life. Our experience with patients who have developed epiphora suggests that ophthalmological examination and careful monitoring during docetaxel treatment may lead to successful management and reduced ocular toxicity.

## Conclusion

In conclusion, it is important for oncologists to be aware of this adverse event, and ophthalmologists should be consulted in cases in which tears appear during docetaxel therapy.

## Consent

Written informed consent was obtained from the patients for publication of this Case Report and any accompanying images. A copy of the written consents is available for review by the Editor-in-Chief of this journal.

## Competing interests

Dr. Takigawa was paid an honorarium from Sanofi-Aventis, Japan for lecturing.

## Authors’ contributions

TY and NT participated in the design of the manuscript. TY, NT, HY and NO followed the patient and drafted the manuscript. HT carried out ophthalmology management. All authors read and approved the final manuscript.
